# Using the PRECEDE-PROCEED model for an online peer-to-peer suicide prevention and awareness for depression (SPAD) intervention among African American college students: experimental study

**DOI:** 10.15171/hpp.2018.02

**Published:** 2018-01-07

**Authors:** Ledetra Shanta Bridges, Manoj Sharma, Jung Hye Sung Lee, Russell Bennett, Sarah G. Buxbaum, Jacqueline Reese-Smith

**Affiliations:** ^1^Behavioral & Environmental Health, School of Public Health, Jackson State University, Jackson, MS 39213, USA; ^2^Epidemiology & Biostatistics, School of Public Health, Jackson State University, Jackson, MS 39213, USA; ^3^Health Policy & Management, School of Public Health, Jackson State University, Jackson, MS 39213, USA; ^4^Psychology, College of Liberal Arts, Jackson State University, Jackson, MS 39217, USA

**Keywords:** Theory, Program, Mental health

## Abstract

**Background:** Suicide rates are high among African American students because they are at a greater risk of depression. A commonly used suicide prevention approach is the gatekeeper training. However, gatekeeper training is neither evidence-based nor has it been identified as culturally-appropriate for African American college students. Therefore, the purpose of this study was to develop and evaluate an online peer-to-peer PRECEDE-PROCEED model based depression awareness and suicide prevention program that was culturally appropriate for African American college students.

**Methods:** The setting was a predominantly Black institution in southern USA. A pre-experimental repeated measures one group design was used to measure changes in peer educators’ (n = 29) predisposing factors regarding knowledge, skills and attitudes pertaining to depression, reinforcing factors or receiving support from peers, healthcare professionals and teachers to help someone with depression, enabling factors or sureness of finding organizations to help someone with depression, and behavior for helping someone with depression at pretest, posttest and 1-month follow-up. A posttest only one group design was also used to measure effect on predisposing factors and behavior of students (n = 300) trained by peer educators.

**Results:** There were statistically significant improvements in attitudes related to depression as disease (P = 0.003; η^2^ = 0.39), attitudes about managing depression (P = 0.0001; η^2^ = 0.30), skills(P = 0.0001; η^2^ = 0.41), reinforcing factors (P = 0.018; η^2^ = 0.13), enabling factors (P = 0.0001;η^2^ = 0.31), and behavior (P = 0.016; η^2^ = 0.14). Changes in knowledge about depression and attitudes about helping people with depression were not statistically significant over time for peer educators. The peer-to-peer training was not completely effective in transferring corresponding changes for students trained by peers.

**Conclusion:** The program was effective for peer educators but peers could not significantly influence other students in all domains. This study provides a starting point toward evidencebased approaches for health promotion interventionists addressing depression awareness and suicide prevention among African American college students.

## Introduction

Depression and suicide are 2 global public health concerns. Suicide is the second leading cause of death among 15-29-year-olds globally,^1^ with an alarming rate among African-American college students. According to the Suicide Prevention Resource Center,^2^ suicide is the third leading cause of death for young Black males between the ages of 15–24 years. According to United States’ Substance Abuse and Mental Services Health Administration’s (SAMSHA’s) 2013 report, men and women between the ages of 18 and 25 years had the highest percentage of major depressive episodes.^3^ Similarly, African-American college students have also been described as having a greater risk for depression compared to other groups. Dzokoto and colleagues^4^ contend that African-American students are at a greater risk for depression due to factors relating to family, financial, and academic stressors.

Researchers report that there is no single agreed upon risk factor, rather, a myriad of risk factors contribute to the suicide, including stressful circumstances; depression and other mental health disorders; family history of suicide, mental health disorder, or substance abuse; individual characteristics such as low self-esteem, risky behaviors, and loneliness; access to lethal methods^5,6^ school factors such as limited access to resources, stigma, negative social and emotional environment, discrimination, and media; and lack of parental support.^7^ The psychosocial risk factors affect endophenotypes and clinical endpoints. Specifically, the presence of depressive symptoms increases the likelihood for heart attacks, stroke, end-stage renal disease, retinopathy, and complications in diabetes.^8^

Programs and interventions have been used to manage depression and suicide among college students. Among suicide programs, gatekeeper training programs are commonly used peer-to-peer interventions.^9^ Gatekeeper trainings for suicide prevention consist of a brief educational program designed to teach specific groups of people who are in close contact with a community of individuals the warning signs of a suicide crisis, how to respond to someone who may be displaying suicide warning signs and behaviors, and to refer the at-risk individual to resources and treatment services. The Suicide Prevention Resource Center (SPRC)^10^ identifies some gatekeeper training programs including: “At Risk for University and College Faculty,” “Be a Link,” “Campus Connect,” Question, Persuade, and Refer (QPR).” The QPR Gatekeeper training program was developed by Dr. Paul Quinnett in 2007 and is an approach where gatekeepers are trained and the trained gatekeepers then train others to widen a community of trained individuals.^11^

Some other well-known global gatekeeper training interventions are: Sources of Strength (SOS), Applied Suicide Intervention Skills Training (ASIST) by Living Works and Yellow Ribbon International (YR) for Suicide Prevention.^12^ The SOS is a universal suicide prevention program designed to build socioecological protective influences among teens and young adults to lessen the likelihood that vulnerable individuals will become suicidal. In the SOS program, peer leaders are trained to deliver hope, help, and strength messages to individuals who are at risk for suicide. ASIST is an international suicide prevention program that was designed as “suicide first-aid.” ASIST’s purpose is to increase the adequacy of crisis center counselors’ suicide risk assessments and interventions, and decrease callers’ crisis and suicide states. YR is a national and international suicide prevention program designed to prevent youth and young adult suicide through education, training, and public awareness campaigns. The training includes discussing the history of Yellow Ribbon; adolescent suicide (including statistics, myths, and warning signs); and the importance of seeking help from an adult for yourself or for a suicidal peer.

While little experimental research on the efficacy and effectiveness of gatekeeper training programs exists, gatekeeper training has been claimed to be somewhat effective in enhancing knowledge^13^; skills, and self-efficacy.^9^ Wyman et al,^14^ found the SOS program to be effective in enhancing protective factors among peer leaders trained to implement messaging among high school students. Research evaluating YR found that student help-seeking did not increase following the training; however, help-seeking with calling the suicide hotline did increase.^15^

Much of the research has primarily focused on suicide. However, many gaps exist in the literature. First, programs addressing both suicide and depression are scarce. Second, while many of the suicide prevention programs and interventions such as the gatekeeper trainer approach have proven somewhat efficacious with knowledge transfer, behaviorally robust, evidence-based suicide prevention and depression awareness interventions are scarce. Third, the efficacy of gatekeeper training has not been empirically demonstrated with behavior change.^16^ Despite the high risk of suicide among African-Americans, few, if any suicide prevention and depression awareness programs exists that are culturally specific to the population. In fact, research by Cimini et al,^17^ reported that none of the studies had examined the effects of gatekeeper training. Cimini et al^17^ also reported that the content of such training was not designed to be relevant to the unique needs, cultures, and concerns of specific minority groups. Despite the high risk of suicide and depression among African-Americans, few if any suicide prevention and depression awareness programs exist that are culturally specific to the population. Lastly, there have not been any reported online interventions of peer-to-peer depression awareness and suicide prevention programs.

In light of the above criticisms, to strengthen health promotion interventions for depression awareness and suicide prevention, this study entailed the development, implementation, and evaluation of an evidence-based, peer-to-peer; online intervention using the PRECEDE-PROCEED model. The PRECEDE-PROCEED model^18^ is a well-established framework for planning a health promotion program. The model presents a guide for applying theories and concepts in the development and evaluation of health education programs.^18^ The online culturally-appropriate peer-to-peer education program was specifically designed for African-American students. The study aimed at identifying the effects of the PRECEDE PROCEED model based “Suicide Prevention and Awareness for Depression (SPAD)” program on changing peer educators’ predisposing factors (knowledge, attitude, and skills), reinforcing factors, enabling factors, and behavior from before to immediately after completion of the training to one month following the intervention. The study also examined the effect of peer educators training on predisposing factors (knowledge, self-reported skills, attitudes) and behavior of trained African-American students regarding depression and suicide following the brief peer-to-peer training.

## Materials and Methods

### Design

There were 2 designs used in this study. The first design was a pre-experimental repeated measures one group design. Data in this section measured changes in peer educators’ predisposing factors (knowledge about depression, attitudes about disease of depression, attitudes about managing depression, attitudes about helping people with depression, and skills about helping people with depression) reinforcing factors, enabling factors, and behavior at 3 different points in time: pretest, posttest, and one-month follow-up. The second design was a one group posttest only design. Data in this section measured peer trained students’ knowledge about depression, attitudes about disease of depression, attitudes about managing depression, attitudes about helping people with depression, and skills about helping people with depression, and behavior following the brief depression awareness and suicide prevention training.

### Population and sampling

**Figure 1:**
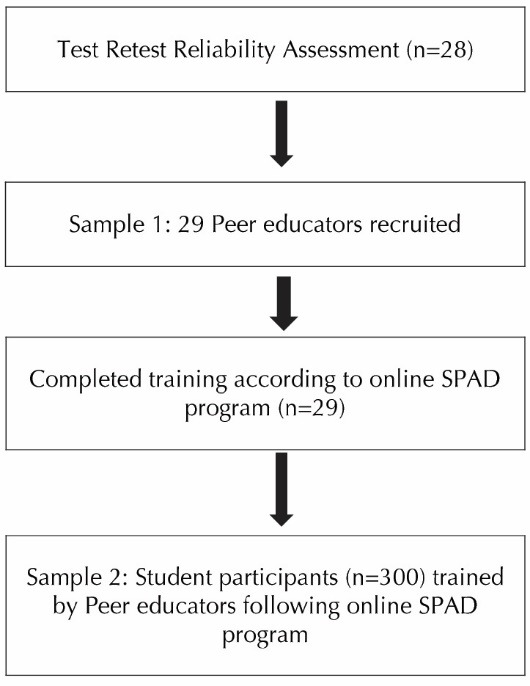
Sample recruitment and training process.

The target population for this study was African-American college students between the ages of 18 to 24 years in the United States. A southern, predominantly Black, university was selected for the intervention as it provided the demographics of the population and convenience needed to evaluate the program. This study involved 2 samples. The first sample consisted of 29 peer educators. The sample size was calculated using G*Power^19^ with an alpha of 0.05, power of 0.80, effect size of 0.25, number of groups as one, number of measurements as 3, correlation among repeated measures as 0.5 and non-sphericity correlation (epsilon) as one that yielded the sample size to be 28 which was then inflated by 5% to adjust for any missing data. Peer educators were the selected student leaders who were trained according to the online culturally-appropriate SPAD program to recognize and counsel their peers dealing with emotional distress and suicidal ideations or suicidal behaviors. Peer educators were further required to train other students. The second sample consisted of a sample size of 300 student participants. This sample size was arrived at because each peer educator was required to provide a brief training to 10 or more students, each of whom were then asked to complete a post test. This was a convenience quota sample (Figure 1).

### Instrumentation

A set of instruments, (*a*) a 40-item instrument for peer educators measuring demographics, predisposing factors (knowledge about depression, attitudes about disease of depression, attitudes about managing depression, attitudes about helping people with depression, and skills about helping people with depression), reinforcing factors, enabling factors, and behavior and (*b*) a subset of first instrument consisting of 33 items for students trained by peer educators measuring peer trained students’ demographics, predisposing factors, and behavior were developed. The main instrument was validated for face and content validity by a panel of 6 experts (2 of which were subject experts, 2 were experts on PRECEDE-PROCEED model and 2 were experts in college students) in 2 rounds. The instrument was also administered at a different predominantly Black institution of higher education for test retest reliability assessment (n=28) and all subscale correlation coefficients were found to be acceptable (0.73 to 0.97). Internal consistency on data collected from all students in the study (n=357) revealed Cronbach’s alpha over 0.80 for 5 of the subscales. The 2 subscales that had lower Cronbach’s alpha were still used because these were new subscales. Construct validation on the data collected from all students (n=357) met the criteria of eigenvalues over 1.0 and factor loadings over 0.28 confirming 7 different one factor solutions. Maximum likelihood method for factor extraction was employed. The Scree test for all subscales was satisfied and no rotations were required to improve the interpretability of the factor structures.

### Intervention

Based on the application of 2 nationally recognized textbooks, *Practical Stress Management*^20^ and *Foundations of Mental Health Promotion*^21^ and use of the PRECEDE-PROCEED model for planning, implementation, and evaluation,^18^ a team of 6 panel experts and 3 African-American doctoral students applied the constructs to design and plan the culturally-appropriate SPAD program. The team developed a 4 module culturally-appropriate online depression awareness and suicide prevention curriculum in the Blackboard learning platform.

In module one, peer educators learned about the statistics, signs and symptoms, and risk factors of depression and suicide in the United States and among African-Americans, early detection of depression and treatment, and how to identify and help someone with depression or suicidal ideations/behaviors. In module 2, peer educators learned about the benefits of helping others, self-care to manage stress, and support resources for peer educators. In module 3, peer educators learned about their responsibilities; counseling and psychotherapy skills; relaxation skills; and counseling and psychotherapy resources. In module 4, peer educators learned about on campus and off campus resources. At the end of each module, peer educators had specific tasks to complete which were based on practicing the skills learned in the modules. The modules were designed so that peer educators could have contact with a mental health professional throughout the training. Peer educators were given 4 weeks to complete the online training. Upon completion of the online training, the trained peer educators participated in deploying the program across the institution. They delivered the intervention, helped monitor student trainees, and helped with collecting the posttest questionnaire from the students whom they trained. The peer educators were given one to 2 weeks to deliver the intervention to at least ten of their peers.

### Scoring of the Scales 

Each of the subscales had its scoring scales for peer educators. Please see Table 1. The participants’ knowledge about depression was measured by an 8-item *True* or *False* question. Examples of the items are “Depression can lead to suicide.” The rating scale of the 8 items was summed to achieve a possible range of 0-8 with the higher scores reflecting an increase in knowledge of depression.

The participants’ attitudes about the disease depression, attitudes about managing depression, and reinforcing factors were all independently measured by a 3-item Likert-type self-reporting rating scale. An example of the items for attitudes about the disease depression is “Depression is a serious disease.” An example of the items for attitudes about managing depression is “Depression is treatable.” An example of the items for reinforcing factors is “How sure are you that you will receive support from your peers to help someone with depression?” The rating scales of each of the 3 items was summed to achieve a possible range of 0-12 with the higher scores reflecting increase in attitudes about the disease depression/attitudes about managing depression/ reinforcing factors.

The participants’ attitudes about helping people with depression, skills about helping people with depression, and enabling factors were all measured by a 4-item Likert-type self-reporting rating scale. An example of the items for attitudes about helping people with depression is “I would be willing to talk to someone suffering from depression about their problem.” An example of the items for skills about helping people with depression is “How sure are you that you can identify someone with depression?” An example of the items for enabling factors is “How sure are you that you will be able to find an organization/a faith-based organization to help someone with depression?” The rating scale of the 4 items was summed to achieve a possible range of 0-16 with the higher score reflecting an increase in attitude about helping people with depression/skills about helping people with depression/enabling factors.

The participants’ behavior was measured by a 2-item Likert-type self-reporting rating scale. An example of the items is “How likely is it that you will help someone with depression.” The rating scale of the 2 items was summed to achieve a possible range of 0-8 with the higher score reflecting an increase in behavior of helping someone with depression. Each of the subscales had its scoring grade for students. See Table 2.

### Process evaluation

Process evaluation took place with the peer educators upon completion of the training. The process evaluation was calculated based on attendance of the program, online platform reports, and satisfaction survey of the peer educators. The content of the modules, design of the modules, activities of the modules, facilitation by the peer educators, pace of the course, learning from the course, and the usefulness of the course were measured.

### Data collection

Peer educators were asked to complete the instrument on 3 occasions: at baseline, upon completion of the training (posttest) and at one-month follow-up. The questionnaires were matched. Students trained by peer educators completed a one-time posttest only survey following the brief training provided by the peer educators. This survey was administered either via online survey software or face-to-face. All data collected from participants were kept confidential. The participants were informed that they could withdraw their consent to participate in the study without consequences at any time. They also had a choice to not answer any questions they did not feel comfortable answering. The peer educators received a $100 gift card for participation upon completion of the one-month follow-up.

### Data analyses

All data were analyzed using the Statistical Package for the Social Sciences (SPSS) version 23. Descriptive statistics were used to describe the demographic characteristics and study variables of the sample. The means, standard deviations and ranges for metric variables and frequencies and percentages for categorical variables were computed for both peer educators and students trained by peer educators. For inferential statistics repeated measures analyses of variance (ANOVA) were used to report changes in knowledge, skills, attitudes, reinforcing factors, enabling factors, and behaviors of depression and suicide prevention strategies pre-training, post-training and one month following the training for the peer educators. Binominal tests were used to investigate whether the proportion of students with satisfactory knowledge, skills, attitudes, and behaviors differed from those with unsatisfactory score range on predetermined cut-offs.

## Results

The recruitment for the study began in April 2016 and continued until August 2017. For the first stage of the study that of peer educator training a total of 107 participants were recruited over that time period from which 29 finished all the requirements including providing the training to other students. For the second stage of the study a quota sample of 300 students trained through peers was established with a minimum of 10 fellow students trained by each trained peer. This number was reached in August 2017 through 29 peer educators when the recruitment was stopped. A summary of the demographic variables for the peer educators and students trained by the peer educators are depicted in Table 3. The mean age of the peer educators was found to be 19.9 years and 20.6 years for the trained students, with a minimum age of 18 years and maximum age of 24 years for both samples. For the peer educators, majority of the students were females (82.8%), lived on campus (58.6%), and did not work (62.1%). For the trained students, more than half of the students were females (64.3%), lived on campus (83.3%) and did not work (60%). For the peer educators, more than half of the students were either freshmen (37.9%) or seniors (27.6%), while 17.2% were either sophomores/juniors. For the trained students, a more than half of the students were freshmen (53.3%) while 18.3 percent were seniors, 15% were juniors, 12 percent were sophomores and 1.3% were graduate students. Four peer educators or 13.8% reported ever being diagnosed with depression while 25 or 86.2% reported never been diagnosed with depression. And there were 5.3% of trained students who had ever been diagnosed with depression and 6.3% of students who were currently suffering from depression. One peer educator reported currently suffering from depression while 28 (96.6%) reported not currently suffering with depression.

A summary of the descriptive statistics of the study variables for the peer educators have been described in Table 4. A total of 8 study variables were evaluated. The mean scores and standard deviations were reported from pretest to posttest to 1-month follow-up. A marked improvement in mean scores took place in all variables except attitudes about the disease depression from pretest to posttest to 1-month follow-up. The mean score of the peer educator’s attitudes about the disease depression increased from 8.17 units at baseline to 9.34 units upon completion of the online training and gradually decreased after posttest to 8.82 units at 1-month.

Summary of repeated measures ANOVA of all variables are reported in Table 4. Attitudes about the disease depression, attitudes about managing depression, skills about helping people with depression, reinforcing factor, enabling factors, and behavior were statistically significant and increased with training. However, knowledge about depression and attitudes about helping people with depression were not significantly significant.

Process evaluation was measured on a scale of poor (0), fair (1), good (2), very good (3), and excellent (4) measuring the content of the modules, design of the modules, activities of the modules, facilitation by the peer educators, pace of the course, learning from the course, and the usefulness of the course. The peer educators rated the modules very good and excellent, identifying knowledge and facts about depression/suicide and resources that were presented as strength. They asked for more information/videos about depression and a need to improve the operation of the online platform.

A summary of descriptive and binominal statistics of the students trained by peer educators performance is provided in Table 5. There were significant differences in the proportion of students with satisfactory scores and unsatisfactory scores trained by peer educators for all variables. For the variable attitudes about the disease depression, 57% of students scored satisfactory while 43% scored unsatisfactory and for attitudes about helping people with depression about 86% scored satisfactory and only 14% scored unsatisfactory.

For remaining variables, more students scored unsatisfactory as compared to satisfactory.

## Discussion

The purpose of this study was to develop, implement, and evaluate a PRECEDE-PROCEED model based depression awareness and suicide prevention program that was culturally appropriate for African-American college students. To evaluate the intervention, valid and reliable instruments were used to measure predisposing factors (knowledge, attitudes, and skills), reinforcing factors, enabling factors, and behavior for the peer educators. The predisposing factors and behavior were measured for students trained by the peer educators following the intervention.

### Peer Educators Training

The results revealed that the program was effective in improving predisposing factors, reinforcing factors, enabling factors, and behavior for the peer educators. The results indicated that peer educators had some knowledge about depression at baseline. This could be due to peer educators’ exposures to campus activities, the Internet, depression in family or friends, and/or some classroom discussions about this topic area. The slight improvements in mean scores overtime indicate that peer educators, once having been exposed to this topic, continued to gain knowledge about depression. The slight increase in knowledge could be attributed to the intervention that attempted to build on the student’s insight by educating them in a manner that was culturally specific. The baseline results indicate that peer educators viewed the disease of depression as a problem. A plausible explanation perhaps is related to individual awareness of depression and the negative stigma associated with mental illness. A reason that there was an increase in attitudes about the disease depression following the training could be the knowledge acquired and the specific attitudinal change teachings carried out in the training. No previous study has measured knowledge about helping people with depression specifically among African-American college students. However, a study that evaluated a statewide prevention and awareness training similarly found an increase in participant’s knowledge and efficacy to help someone who is suicidal.^22^ Another study revealed that e-learning gatekeeper modules improve knowledge and self-confidence regarding suicide prevention.^23^

The results indicate that many peer educators’ attitudes about managing depression were high at baseline. This could be due to their beliefs of relying on medical professions and medical science. However, many of the students reported that they had never been diagnosed with depression thus making managing depression not personally applicable to many. The increase in attitudes about managing depression at posttest can be explained by the activities of the training program and skills acquired regarding managing depression upon completion of the training. Peer educators in the study reported a high score regarding attitudes about helping people with depression at baseline inclining that these students were self-motivated to begin with and did not necessarily need any training in this regard. The high scores continued posttest and at 1-month follow-up, reflecting a slightly small increase, suggesting that the African-American peer educators in this study were already willing to help someone with depression at baseline and remained willing to help someone with depression over time which could be culturally related to African-American students’ willingness to support one another. A possible reason that there was an increase in attitudes about helping people with depression could be the educational activities that were provided and the resulting skills that were acquired upon completion of the training. No previous study measures this construct regarding depression/suicide among African-American college students. However, a study that evaluated a suicide prevention training for helpers found an increase in their attitudes and skills.^24^

The results indicate that peer educators reported having some skills about helping people with depression at baseline. One reason that there was a steady increase in skills over time was that the students completed educational activities and exercises completed in the modules. The exercises required students to practice their communication skills and relaxation techniques. There was no previous study found that addressed changes in skills about helping people with depression among African-American college students. A study that was conducted by Cross et al^13^ revealed that gatekeeper training was effective in increasing training skills. Another study showed that the gatekeeper training increased crisis communication skills and suicide related knowledge for college campus resident advisors.^25^ Pasco et al,^9^ revealed that gatekeeper training was effective in increasing self-reported comfort in asking about suicide. A study that evaluated a suicide prevention training for helpers found an increase in their attitudes and skills.^24^

The results of this study indicate that peer educators were sure that they would receive support from others or reinforcing factors over time. The increase for reinforcing factors could be due to cultural beliefs regarding the importance of support among African-Americans. The increase in enabling factors scores could be due to the intervention which provided local, state, and national resources available for the target population. The results revealed an increase in the peer educators’ behavior. A reason for the increase in behavior scores could be due to the corresponding increases in predisposing, enabling, and reinforcing constructs gained in the intervention. Another reason for the increase could be due to the already high scores of the students’ attitudes about helping people with depression. Because there was no report in the literature found examining gatekeeper training and reinforcing factors, enabling factors, and behavior for African-American college students, specific comparisons cannot be made. A study that evaluated a brief gatekeeper training intervention resulted in an increase in reinforcing factors and behaviors.^26^

### Students trained by peer educators

The peer-to-peer training did not add much in terms of improving knowledge about helping people with depression, attitudes about managing depression, skills about helping people with depression, and behavior for most of the students. As it was the peer educators’ responsibility to deliver the information acquired from their training, their training of other students was not closely monitored by the researchers which could have resulted in the large number of students with unsatisfactory scores. It may still be worthwhile to develop guidelines or training manual for peer educators who will train other students and closely monitor the training. While many constructs resulted in more unsatisfactory scores, 2 constructs: attitudes about the disease depression and attitudes about helping people with depression resulted in more students with satisfactory scores. The emphasis of this endeavor was to influence attitudes more than knowledge and that seems to be the reason for improvement in attitudinal scores. While students seemed motivated to help, they may have felt somewhat unprepared because many of the subscales scores did not meet satisfactory thresholds. In the future, peer educators need to be equipped with educational methodological tools to do a better job of imparting the newly acquired predisposing, reinforcing, and enabling constructs knowledge.

### Limitations

There were some limitations of this study. Self-reports were used for data collection in this study to collect pertinent data which pose potential threats of error including participants’ bias and social desirability bias, and potential problems with recall. For this study, convenience sampling potentially introduced sampling bias. The study did not use random selection of subjects, thus not adding confidence in generalization of the results. There was selection bias because students between the ages of 18 and 24 years were only allowed to participate in this study. College students that were younger than age 18 or older than 24 years were not allowed to participate in this study. This study did not use a comparison or control group which future studies should use.

The set of instruments developed for this study (utilizing the PRECEDE-PROCEED model) were developed for the first time. Thus, having a new scale resulted in low Cronbach’s alpha scores for 2 subscales in this study. While we chose to sacrifice internal consistency to some extent in lieu of having solid construct validity in this study, future studies should focus on improving it via adding more items to both the attitudes about the disease depression and attitudes about managing depression subscales.

Further improvement is needed for the instrument. The knowledge construct could be more culturally specific in its operationalization according to the study population. Measuring depression as one item on the questionnaire was difficult, thus making it another limitation of this study. Future studies should measure depression as more than one item while also assessing for the risk of depression. There were some limitations pertaining to the intervention. Giving more details and guidance regarding training techniques by the peer educators to train their peers would have enhanced the intervention.

### Recommendation for research

This study demonstrated that a brief online PRECEDE-PROCEED model based suicide prevention and depression awareness intervention was effective in improving predisposing factors, reinforcing factors, enabling factors, and behavior in a study sample of African-American college students. However, a peer-to-peer brief suicide prevention and depression awareness training was not effective in improving predisposing factors and behavior among a sample of African-American college students. It is recommended that this study be replicated with refinements as discussed earlier to African-American college students in various geographical areas to establish further efficacy of this approach.

Specific recommendation for improving the measurement can be made based on the findings of this study. As discussed earlier, making changes to the measurement instrument to include salient and precise information to the predisposing, enabling, and reinforcing constructs can improve the questionnaire. The present study provides a baseline in which further work can result. Based on this study one may conclude that college students have some insight regarding suicide prevention and depression awareness. Future research should take place to monitor the peer educators’ application of the knowledge and strategies learned via closely coaching and monitoring the peer-to-peer training process.

The PRECEDE-PROCEED based intervention should be compared to a control group. This could include comparing the intervention group to an assessment only intervention group or a knowledge-based intervention group. Such research would provide insights to the impact of the intervention. The intervention could also be compared with another form of suicide prevention and depression awareness, for example the SOS or YR. As stated before, SOS is a universal suicide prevention program designed to build socioecological protective influences among teens and young adults to reduce the likelihood that vulnerable individuals will become suicidal. YR is a national and international suicide prevention program designed to prevent youth and young adult suicide via education, training, and public awareness campaigns. Comparing the intervention to another form of suicide prevention and depression awareness intervention could give detail to which intervention is more effective. The intervention could also be compared to other behavioral theories which could give detail to the advantages and disadvantages of the different theories while also reporting the effectiveness of the theories.

Finally, this study only measured changes 1-month following the intervention. A longer period of follow-up at 6 months could take place if the intervention is conducted at the beginning of the academic school year. Follow-up could also take place one or more years if a freshman or sophomore group is followed until completion of school. Such information can provide the long-term impact of the intervention.

### Implications for practice

The findings of this study have important implications for health promotion practitioners who are developing an instrument based on the PRECEDE-PROCEED model and implementing a brief theory-based intervention for suicide prevention and depression awareness. The duration of the training was 4-modules and presented online, making it easily accessible for health education professionals in public health settings who work with college students. This study reaffirms the findings of previous studies that gatekeeper trainings are successful in improving gatekeeper training skills. In this study, the baseline scores for the constructs were high for peer educator training, making demonstrating a statistically significant modification in scores of 2 subscales (knowledge about depression and attitudes about helping people with depression) more challenging due to the intervention. All other subscales demonstrated a statistically significant difference for peer educator training. However, the improvements in predisposing, enabling, and reinforcing constructs for peer educator training did not translate to significant improvements in all domains for the students trained by peer educators. The PRECEDE-PROCEED model is a very broad planning model that does not provide specific guidance for influencing behaviors as to which specific predisposing, enabling, and reinforcing factors need to be prioritized. This made the training very diffuse. Recent advancements in health behavior research have proposed models based on multiple behavioral theories that prioritize salient constructs. One such model is the multi-theory model (MTM) of health behavior change.^27,28^ This model breaks down behavior change into initiation and sustenance steps. For the initiation model, behavioral confidence is a salient predisposing factor, participatory dialogue of advantages outweighing disadvantages is a salient reinforcing factor, and changes in physical environment is a salient enabling factor. Similarly, in the sustenance model, emotional transformation is a salient predisposing factor, practice for change is salient reinforcing factor and social environment is a salient enabling factor. Thus, the MTM of health behavior change is a more focused and precise approach for designing behavioral interventions. Future interventions must embody the multi-theory model or similar multiple theory approaches.

## Conclusion

Based on the results of this study, it can be concluded that a PRECEDE PROCEED Model based SPAD improved peer educators’ predisposing factors, reinforcing factors, enabling factors, and behavior over time in a sample of African-American college students. There were no statistically significant differences in knowledge scores and attitudes about helping people with over time. However, all the constructs, except knowledge, showed improvements in mean scores over time. It can further be concluded that the peer educators training did not have a statistically significant effect on the knowledge, self-reported skills, attitudes, and behavior of African-American students regarding depression and suicide following the brief peer-to-peer training. However, attitudes about the disease depression and attitudes about helping people with depression were the only 2 constructs in which majority of the students obtained a satisfactory score.

This study provides additional information for behavioral interventions addressing suicide prevention and depression awareness among the African-American college students. This is the first research study in which an attempt was made to develop an evidence and theory-based peer-to-peer depression awareness and suicide prevention program. Future interventions must refine this approach using newer behavioral theories developed in the field.

## Ethical approval

Permission was obtained from the IRB at the Jackson State University dated April 1, 2016 to conduct both phases of this study. All participants were provided information about the study and were asked to sign an informed consent form before participating in the study. .

## Competing interests

The authors declare that they have no competing interests.

## Authors’ contribution

LB conceptualized the study, validated the instrument, developed the intervention, conducted the training, collected the data, analyzed the data, interpreted the data, wrote the first draft of the manuscript and helped in finalization of the manuscript. MS conceptualized the study, developed the instrument, helped with intervention development, data analyses, data interpretation and helped finalize the manuscript. JHSL helped with data analyses, data interpretation and finalization of the manuscript. RB helped with data interpretation and finalization of the manuscript. SB helped with data interpretation and finalization of the manuscript. JR helped with intervention development, data interpretation and finalization of the manuscript.

**Table 1:** Summary of peer educators instrument subscales and scoring scales

**Item No.**	**Subscale**	**No. of Items**	**Scoring scale**
Q1-Q7	Demographics	8	
Q8-Q9	Depression History	2	
Q10-Q17	Knowledge about Depression	8*	0 – 8
Q18-Q20	Attitudes about the Disease Depression	3	0 – 12
Q21-Q23	Attitudes about Managing Depression	3	0 - 12
Q24-Q27	Attitudes about Helping People with Depression	4	0 – 16
Q28-Q31	Skills about Helping People with Depression	4	0 – 16
Q32-Q34	Reinforcing Factors	3	0 – 12
Q35-Q38	Enabling Factors	4	0 – 16
Q39-Q40	Behavior of Helping Someone with Depression	2	0 – 8

*True/False questions.

**Table 2:** Summary of students instrument subscales and scoring scales

**Item No.**	**Subscale**	**No. of Items**	**Scoring Scale Pass (p)/Fail (f) Satisfactory**
Q1–Q7	Demographics	8	
Q8–Q9	Depression History	2	
Q10–Q17	Knowledge about Depression	8*	0–8 (*P*= ≥6, f= ≤5)
Q18–Q20	Attitudes about the Disease Depression	3	0–12 Satisfactory ≥9
Q21–Q23	Attitudes about Managing Depression	3	0–12 Satisfactory ≥9
Q24–Q27	Attitudes about Helping People with Depression	4	0–16 Satisfactory ≥12
Q28–Q31	Skills about Helping People with Depression	4	0–16 Satisfactory ≥12
Q32–Q33	Behavior of Helping Someone with Depression	2	0–8 Satisfactory ≥6

*True/False questions, > means equal to or greater than, < means equal to or less than.

**Table 3:** Summary of demographics of peer educators and students trained by peer educators

**Variable**	**Category**	**Peer educators** **Mean±SD or No. (%)**	**Students Trained** **Mean±SD or No. (%)**
Age		19.9±1.84	20.6±1.3
Gender	Male	5 (17.2)	5 (17.9)
	Female	24 (82.8)	23 (81.1)
Race/Ethnicity	Black	29 (100)	28 (100)
Classification in school	Freshman	11 (37.9)	4 (14.3)
	Sophomore	5 (17.2)	7 (25)
	Junior	5 (17.2)	14 (50)
	Senior	8 (27.6)	3 (10.7)
GPA	2.00-2.49	3 (10.3)	1 (3.6)
	2.50-2.99	4 (13.8)	15 (53.6)
	3.00-3.49	11 (37.9)	9 (32.1)
	3.50-4.00	11 (37.9)	3 (10.7)
Living quarters	On campus	17 (58.6)	18 (64.3)
	Off-campus	12 (41.4)	10 (35.7)
Working status	Not Working	18 (62.1)	15 (53.6)
	Working	11 (37.9)	13 (46.4)
Ever diagnosed with depression	No	25 (86.2)	28 (100)
	Yes	4 (13.8)	
Currently suffering with depression	No	28 (96.6)	25 (89.3)
	Yes	1 (3.4)	3 (10.7)

**Table 4:** Summary of descriptive statistics of the study variables for the peer educators trained in the intervention (n=29)

**Variable**	**Pretest** **Mean (SD)**	**Posttest** **Mean (SD)**	**Follow-up** **Mean (SD)**	***P *** **value**	**η** ^ 2 ^
1. Knowledge about depression	4.86 (1.09)	5.28 (0.88)	5.38 (1.01)	0.073	--
2. Attitudes about disease depression	8.17 (1.56)	9.34 (1.23)	8.82 (1.75)	0.003	0.39
3. Attitudes about managing depression	8.66 (1.99)	10.24 (1.27)	10.69 (1.39)	0.0001	0.30
4. Attitudes about helping people with depression	15.34 (1.37)	15.62 (0.98)	15.72 (1.13)	0.259	--
5. Skills about helping people with depression	10.69 (2.79)	13.69 (2.16)	14 (1.96)	0.0001	0.41
6. Reinforcing factors	9.21 (2.40)	10.10 (2.41)	10.38 (2.06)	0.018	0.13
7. Enabling factors	11.83 (3.99)	13.90 (2.44)	14.93 (1.94)	0.0001	0.31
8. Behavior	6.97 (1.50)	7.45 (1.06)	7.59 (0.91)	0.016	0.14

*P value from one-way repeated ANOVA.

**Table 5:** Summary of descriptive and binomial statistics on students performance (n=300)

**Variable**	**Frequency (%)**		***P*** value***
	Satisfactory*	Unsatisfactory**	
Knowledge	65 (21.7)	235 (78.3)	0.0001
Attitudes about the disease depression	171 (57)	129 (43)	0.021
Attitudes about managing depression	132 (44)	168 (56)	0.043
Attitudes about helping people with depression	257 (85.7)	43 (14.3)	0.0001
Skills	99 (33)	201 (67)	0.0001
Behavior	90 (30)	210 (70)	0.0001

* Passing scores; ** Failing scores; *** *P* value from binomial test.
